# Bacterial RecA Protein Promotes Adenoviral Recombination during *In Vitro* Infection

**DOI:** 10.1128/mSphere.00105-18

**Published:** 2018-06-20

**Authors:** Jeong Yoon Lee, Ji Sun Lee, Emma C. Materne, Rahul Rajala, Ashrafali M. Ismail, Donald Seto, David W. Dyer, Jaya Rajaiya, James Chodosh

**Affiliations:** a Department of Ophthalmology, Massachusetts Eye and Ear, Harvard Medical School, Boston, Massachusetts, USA; b Department of Chemistry and Biochemistry, University of Oklahoma, Norman, Oklahoma, USA; c Bioinformatics and Computational Biology Program, School of Systems Biology, George Mason University, Manassas, Virginia, USA; d Department of Microbiology and Immunology, University of Oklahoma Health Sciences Center, Oklahoma City, Oklahoma, USA; University of Zurich

**Keywords:** adenoviruses, commensal, homologous recombination

## Abstract

Adenovirus infections in humans are common and sometimes lethal. Adenovirus-derived vectors are also commonly chosen for gene therapy in human clinical trials. We have shown in previous work that homologous recombination between adenoviral genomes of human adenovirus species D (HAdV-D), the largest and fastest growing HAdV species, is responsible for the rapid evolution of this species. Because adenovirus infection initiates in mucosal epithelia, particularly at the gastrointestinal, respiratory, genitourinary, and ocular surfaces, we sought to determine a possible role for mucosal microbiota in adenovirus genome diversity. By analysis of known recombination hot spots across 38 human adenovirus genomes in species D (HAdV-D), we identified nucleotide sequence motifs similar to bacterial Chi sequences, which facilitate homologous recombination in the presence of bacterial Rec enzymes. These motifs, referred to here as Chi_AD_, were identified immediately 5′ to the sequence encoding penton base hypervariable loop 2, which expresses the arginine-glycine-aspartate moiety critical to adenoviral cellular entry. Coinfection with two HAdV-Ds in the presence of an Escherichia coli lysate increased recombination; this was blocked in a RecA mutant strain, E. coli DH5α, or upon RecA depletion. Recombination increased in the presence of E. coli lysate despite a general reduction in viral replication. RecA colocalized with viral DNA in HAdV-D-infected cell nuclei and was shown to bind specifically to Chi_AD_ sequences. These results indicate that adenoviruses may repurpose bacterial recombination machinery, a sharing of evolutionary mechanisms across a diverse microbiota, and unique example of viral commensalism.

**IMPORTANCE** Adenoviruses are common human mucosal pathogens of the gastrointestinal, respiratory, and genitourinary tracts and ocular surface. Here, we report finding Chi-like sequences in adenovirus recombination hot spots. Adenovirus coinfection in the presence of bacterial RecA protein facilitated homologous recombination between viruses. Genetic recombination led to evolution of an important external feature on the adenoviral capsid, namely, the penton base protein hypervariable loop 2, which contains the arginine-glycine-aspartic acid motif critical to viral internalization. We speculate that free Rec proteins present in gastrointestinal secretions upon bacterial cell death facilitate the evolution of human adenoviruses through homologous recombination, an example of viral commensalism and the complexity of virus-host interactions, including regional microbiota.

## Abstract (precis)

Adenoviruses are common human mucosal pathogens of the gastrointestinal, respiratory, and genitourinary tracts and ocular surface. Here, we report finding Chi-like sequences in adenovirus recombination hot spots. Adenovirus coinfection in the presence of bacterial RecA protein facilitated homologous recombination between viruses. Genetic recombination led to evolution of an important external feature on the adenoviral capsid, namely, the penton base protein hypervariable loop 2, which contains the arginine-glycine-aspartic acid motif critical to viral internalization. We speculate that free Rec proteins present in gastrointestinal secretions upon bacterial cell death facilitate the evolution of human adenoviruses through homologous recombination, an example of viral commensalism and the complexity of virus-host interactions, including regional microbiota.

## INTRODUCTION

Human adenovirus (HAdV), now known to be both a common enteric and respiratory pathogen (1, 2), was first identified in 1953 from a child’s adenoid specimen in organ culture (3, 4). A double-stranded DNA virus with a linear genome of ~35 kb, HAdV segregates by phylogenomics to seven species (A to G), comprising 84 distinct HAdV genotypes. The largest and most rapidly expanding HAdV species is HAdV species D (HAdV-D) with 54 unique genotypes. Numerous HAdV-D types were first discovered in the feces of human patients with AIDS (5–10), and simultaneous coinfection with more than one adenovirus type is common (11, 12). Single nucleotide polymorphisms in HAdV occur uncommonly, even over decades (13), and the 54 viruses within HAdV-D are highly conserved (around 90% at the nucleotide level). However, homologous recombination between viruses within HAdV-D occurs commonly, particularly at transitions from conserved to hypervariable nucleotide sequence where the relatively high GC content of HAdV-D (~56% overall) drops abruptly (14). Remarkably, every fully sequenced HAdV-D shows evidence for at least two prior homologous recombination events among seven, stereotypically hypervariable, gene segments (14). Taken together, these data suggest that adenoviruses can persistently infect the human intestine, where coinfections set the stage for homologous recombination between highly related genotypes.

The nonenveloped adenovirus capsid takes an icosahedral shape with 12 apices, each marked by a trimeric fiber protein with its distal knob and encircled by five, linked penton base proteins. Each individual penton base protein contains two hypervariable loops (HVL1 and HVL2); HVL2 expresses the canonical arginine-glycine-aspartic acid (RGD) moiety critical to integrin-mediated internalization of the virus (15, 16). Structural data (17) shows that after binding of the fiber knob to one of several possible primary receptors on the target cell, viral internalization is mediated through binding of each RGD in the five-sided penton base capsomer to integrins, inducing in turn their aggregation, conformational change, and autophosphorylation to catalyze downstream intracellular signaling. Previous analysis of hypervariable gene segments in 38 HAdV-Ds showed only 14 distinguishable amino acid patterns (proteotypes) (18) for penton base HVL1 and only 10 for HVL2 (14), consistent with homologous recombination at penton base gene segments as a major driver in the ontogeny of new HAdV-D types (19). Because HVL1 and HVL2 are separated by only 123 amino acids and yet their coding regions recombine independently of one another (19), we predicted the existence of a recombination signal in the intervening, relatively conserved area of the genome between the gene segments for these two hypervariable regions.

In bacteria and bacteriophage, a signal for recombination between homologous DNA is the crossover hot spot instigator, or Chi nucleotide sequence. This was first discovered in bacteriophage lambda and then in bacterial DNA and later was shown to mediate recombination between them (20). The Chi sequence in bacteriophage λ and in Escherichia coli (Chi_EC_) is 5′-GCTGGTGG-3′ (21, 22), and its presence induces the exonuclease function of the bacterial RecBCD enzyme (23). The RecA protein of E. coli is then loaded onto unwound single-stranded DNA (ssDNA) by RecBCD to create an ssDNA-protein filament, which invades homologous double-stranded DNA (dsDNA), leading to homologous recombination (24). A conserved Chi sequence in bacterial genera does not exist (25); the repair enzymes that repair dsDNA breaks and mediate homologous recombination also differ in genera. However, RecA has significant homology to eukaryotic Rad51 and its paralogs (26), enzymes that repair dsDNA breaks in human cells, and facilitate homologous recombination in the human genome (27). Also, the adenovirus and bacteriophage PRD1 exhibit striking structural similarities consistent with a common ancestor (28), suggesting the possibility that mechanisms of phage evolution have survived in the adenoviruses.

These disparate observations led us to consider whether the presence of intestinal bacterial flora during adenovirus coinfection might facilitate homologous recombination and evolution of enteric HAdV-Ds. Evidence for transkingdom interactions between bacteria of the human gut microbiome and enteric viruses has been accumulating recently. In enteric infection with certain RNA viruses, virus hijacks surface bacterial glycans of normal bacterial flora to gain entry to intestinal epithelial cells (29–33). Recently, data suggesting that several enteric bacteria can promote recombination during poliovirus infection have appeared (34). As another common enteric agent, adenoviruses are regularly shed in feces of humans and nonhuman primates for months to years after infection (35–38), suggesting that adenoviruses might also exploit enteric bacterial flora to its own advantage. In the work described herein, we utilized a previously published database of 38 whole HAdV-D genomes (14) to analyze the GC/AT transition zone at the 5′ end of penton base HVL2. We show the presence of Chi-like sequences at the exact juncture of conserved and hypervariable sequence and further show an increase in homologous recombination in the presence of bacterial RecA protein. These data demonstrate a means by which bacterial flora can facilitate genetic exchange between adenoviruses.

## RESULTS

**FIG 1:**
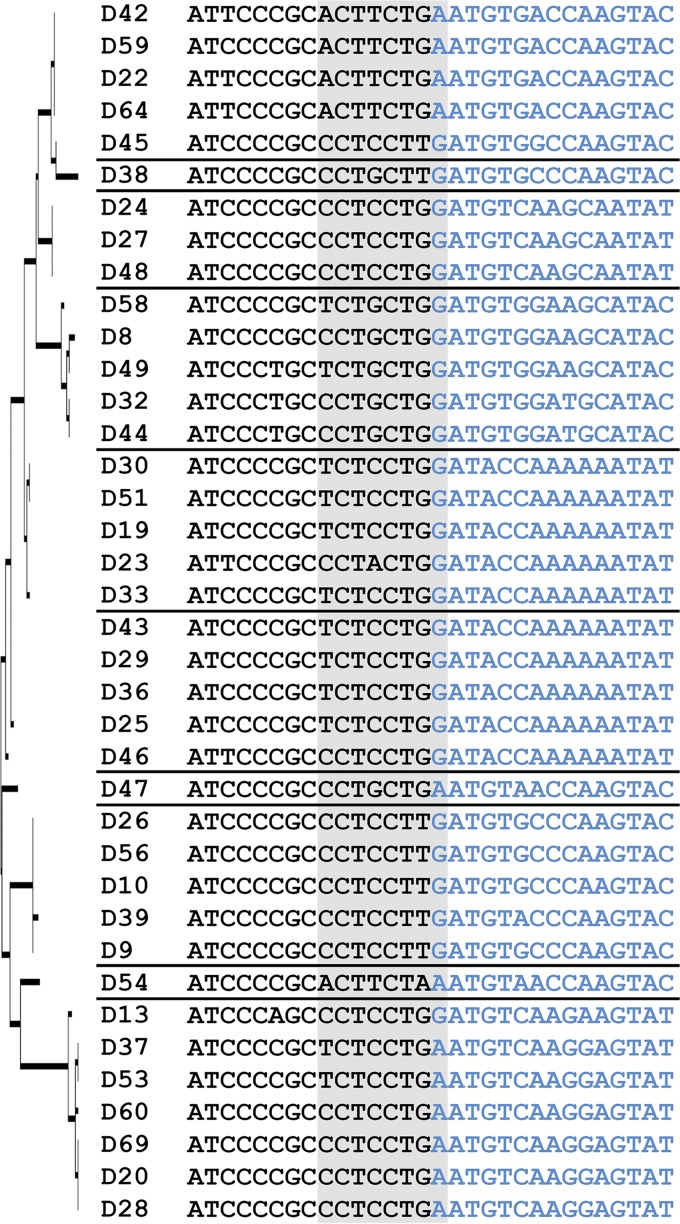
Chi (Chi_AD_) nucleotide sequences in human adenovirus species D (HAdV-D). Thirty-eight HAdV-D genomes were aligned by maximum likelihood analysis, a tree (left) was built based on the amino acid sequences of penton base hypervariable loop 2 (HVL2), and the HAdV-D genomes were divided into proteotypes as previously described (14), shown by a horizontal line separating the virus type names. The 30 nucleotides shown include the junction between conserved nucleotide sequence (black) and HVL2 nucleotide sequence (blue), with 15 nucleotides on either side of the junction. The Chi_AD_ motifs (shown on gray background) fall predominantly within the conserved sequence but include one nucleotide within the hypervariable sequence.

We began by searching the penton base genes of HAdV-D for Chi nucleotide sequences that, in bacteria and bacteriophage, act as signals for recombination between homologous DNA (20). By careful inspection, we identified Chi-like (Chi_AD_) sequences, for example, 5′-TCTCCTGA-3′ in HAdV-D37, in the relatively conserved region immediately 5′ to HVL2 (Fig. 1). We also noted that the nucleotide sequences of putative Chi_AD_ were generally conserved within proteotypes but not between them. In other studies, patterned alterations of GC content in multiples of 15 nucleotides were shown to facilitate homologous recombination between adjacent brome mosaic virus ssRNAs through the formation of hairpin loops in the RNA (39, 40). Our computational analysis of similar GC content transitions across whole HAdV-D genomes showed comparable patterns of GC/AT transition at predicted recombination hot spots around hypervariable gene segments in the three major capsid genes—the same segments that constitute the molecular identity of each virus (14, 19, 41–43)—including the region containing Chi_AD_ immediately 5′ to penton base HVL2 (see Fig. S1 and Table S1 in the supplemental material). We applied mFold (http://unafold.rna.albany.edu/?q=mfold/dna-folding-form) to model the secondary structures of ssDNA surrounding and including Chi_AD_, within the GC/AT transition zone at HVL2. The structures were highly similar within proteotypes, less so between proteotypes (Fig. S2), suggesting that patterned alterations in GC content generate secondary structures that facilitate homologous recombination between HAdV-D types sharing the same Chi_AD_.

10.1128/mSphere.00105-18.1 FIG S1  GC/AT transition zones in the major capsid genes. Low-resolution overview of viruses (38 viruses) from HAdV-D aligned by maximum likelihood analysis and 30-, 45-, and 60-nucleotide GC/AT transition zones identified by a computer algorithm designed to identify >10% change in GC content (14). (A) Penton base gene; (B) hexon gene; (C) fiber gene. Nucleotide positions are shown on the *y* axis, and viruses are shown on the *x* axis. For each gene, the lighter colored region shows the entire gene, and the darker region shows hypervariable regions. There are two hypervariable regions in the penton base gene, two in the hexon gene, and one in the fiber gene. Adenovirus types grouped in the same proteotypes are distinguished by a vertical gap. Hatched lines shows the location of GC/AT transition zones; each black line represents 15 nucleotides. Download FIG S1, PDF file, 2.8 MB. Copyright © 2018 Lee et al. 2018 Lee et al. This content is distributed under the terms of the Creative Commons Attribution 4.0 International license.

10.1128/mSphere.00105-18.2 FIG S2  Predicted ssDNA secondary structures. Penton base sequences for HAdV-D (38 viruses) were aligned by maximum likelihood analysis, and a tree (left) was built based on HVL2. The putative ssDNA (coding strand) secondary structures modeled by mFold for 80 nucleotides around the GC/AT transition zone marking the change from conserved sequence 5′ to HVL2 and the beginning of HVL2, and including the Chi_AD_ motifs (in red) are shown. The lowest free energy structures are shown. Black horizontal lines delineate proteotypes. The three structures to the right are for those viruses in proteotypes with only one member. Download FIG S2, PDF file, 1.6 MB. Copyright © 2018 Lee et al. 2018 Lee et al. This content is distributed under the terms of the Creative Commons Attribution 4.0 International license.

10.1128/mSphere.00105-18.5 TABLE S1  PCR primers and nucleotide sequences. Download TABLE S1, DOCX file, 0.02 MB. Copyright © 2018 Lee et al. 2018 Lee et al. This content is distributed under the terms of the Creative Commons Attribution 4.0 International license.

**FIG 2:**
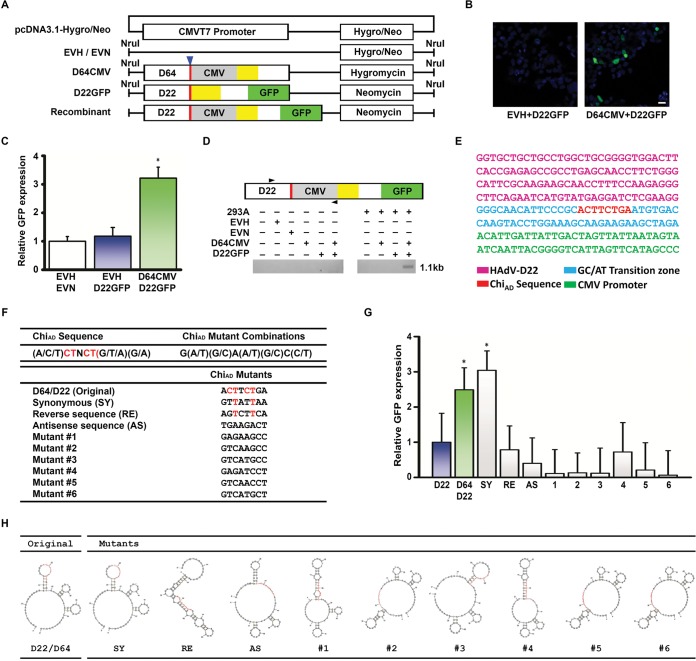
Modeling homologous recombination through Chi_AD_. (A) Constructs for *in vitro* recombination were generated on the pcDNA3.1-hygromycin/neomycin by insertion of either the penton base gene of HAdV-D64 while retaining the hygromycin resistance gene or of HAdV-D22 with coding sequence for green fluorescent protein (GFP) without ATG site, no CMVT7 promoter, and retaining the neomycin resistance gene. HVL2 (yellow boxes), GC/AT transition zone (red boxes with blue arrowhead above), CMVT7 promoter (gray boxes), and GFP open reading frames (green boxes) are indicated. EVH and EVN are hygromycin and neomycin empty vectors minus their CMVT7 promoters, respectively. Modified vectors were linearized with NruI prior to transfection. (B) Fluorescence microscopy for GFP expression in transfected 293A cells show green signal indicating recombination only when both HAdV-D sequence constructs are cotransfected (right micrograph). Original magnification, ×40. Bar, 25 µm. (C) Fluorescent signal graphically represented relative to GFP with cotransfection of an empty vector, demonstrating approximately threefold increase in signal upon cotransfection of Chi_AD_-containing constructs. The value that is significantly different (*P* < 0.05) by ANOVA is indicated by an asterisk. (D) Conventional PCR performed on transfected 293A cells with primer pairs designed to amplify only the hypothesized recombinant (forward primer from HAdV-D22 and a reverse primer for the CMVT7 promoter) shows a band only when both HAdV-D sequence constructs are cotransfected and at the predicted 1.1-kb size. (E) Sanger sequencing of PCR product (from panel D) demonstrating the specific nucleotide sequence predicted for the recombinant construct. (F) A consensus Chi_AD_ sequence was generated *in silico* from 38 HAdV-Ds, and sequences not seen in known viruses were then generated, including D64/22 synonymous (SY), reverse (RE), and antisense (AS) mutants, and six randomly chosen mutants. The consensus nucleotides remaining in the chosen mutants are shown in red. (G) Fluorescent signal relative to GFP expression upon cotransfection of empty vector and D22GFP show recombination with the constructs containing native Chi_AD_ and also with a mutant (from panel F) containing synonymous changes to Chi_AD_ (SY) (*, *P* < 0.05 by ANOVA). Each experiment was performed in triplicate and repeated three times. Error bars represent standard deviations of the means. (H) Secondary ssDNA sequences as predicted by mFold, showing that the original Chi_AD_ and SY mutants, which each recombined in panel G, have highly similar predicted secondary structures.

To examine the determinant(s) of homologous recombination among HAdV-Ds and specifically the role of Chi_AD_, we generated constructs with the Chi_AD_-containing GC/AT transition zones from the penton base genes of HAdV-D22 and -D64, both from the same HVL2 proteotype (14), the former with the green fluorescent protein (GFP) gene without a promoter (D22GFP), and the latter with a CMVT7 (CMV stands for cytomegalovirus) promoter inserted (D64CMV) (Fig. 2A). In 293A cells, cotransfection of linearized D64CMV and D22GFP constructs resulted in GFP expression (Fig. 2B and C). By PCR with primers specific to the recombinant product (Table S1) and subsequent sequencing, we confirmed that the two constructs had indeed recombined at the expected location (Fig. 2D and E), the same recombination locus often seen in HAdV-D recombination in nature (19).

Prior work suggested that a single nucleotide change in Chi can reduce homologous recombination events (44), with particular emphasis on the importance of the thymidine (T) at the third position (45, 46). We generated targeted mutations in both constructs to test the specificity of Chi_AD_ at the single nucleotide level, while maintaining homology between constructs for cotransfections. Chi_AD_ nucleotide identities were determined for HVL2 in each of 38 HAdV-Ds to determine a consensus sequence (Fig. 2F). Mutants not found in any known virus were generated, and nine were selected for testing, including one with nucleotide changes synonymous to D64/22 (SY), one with reverse sequence (RE), one with antisense sequence (AS), and six randomly selected. Mutating Chi_AD_ reduced homologous recombination between vector constructs except for the SY mutant (Fig. 2G). Subsequent mFold analysis showed that the predicted secondary structure for the SY construct and the location of Chi_AD_ in that secondary structure were highly similar to those of the parent Chi_AD_ (Fig. 2H). None of the other predicted structures for Chi_AD_ mutants were similar. The RE mutation, which maintained T at the third position and was expected to undergo homologous recombination, had a predicted secondary structure distinctly different from that of the naturally occurring Chi_AD_. Therefore, nucleotide sequence and secondary structure may be considered covariant determinants of homologous recombination.

**FIG 3:**
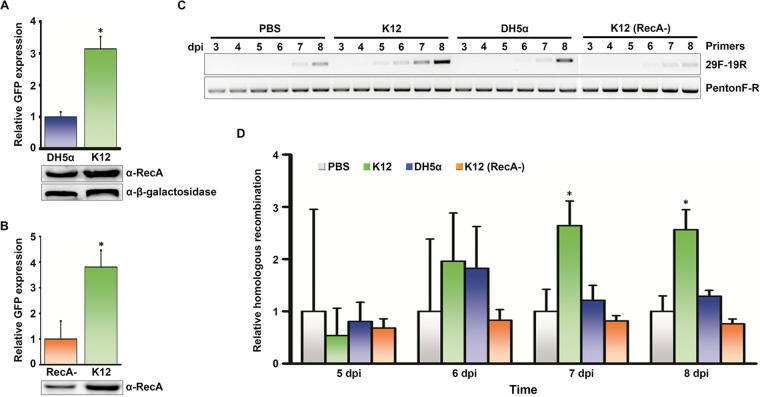
Promotion of homologous recombination by bacterial lysate in intestinal epithelial cells. (A) Quantification of GFP expression by Chi_AD_-containing constructs in the presence of a lysate of E. coli K-12 strain or its RecA mutant DH5α demonstrate reduced recombination in 293A cells pretreated with RecA mutant (*, *P* < 0.05 by Student’s *t* test). The Western blot below the graph shows expression of RecA in both E. coli lysates and β-galactosidase loading control. α-RecA, anti-RecA antibody. (B) Expression of GFP in cotransfected 293A cells pretreated with either *E*. *coli* K-12 lysate depleted of RecA (RecA-) by immunomagnetic beads, or unmodified K-12 lysate, showing reduction of recombination when RecA is depleted (*, *P* < 0.05 by Student’s *t* test). Western blot of depleted and native lysates is shown. (C) Conventional PCR performed on C2BBe1 cells pretreated with either PBS, *E*. *coli* K-12 lysate, DH5α lysate, or RecA-depleted K-12 lysate, and coinfected with HAdV-D19 and HAdV-D29, from 3 to 8 days postinfection (dpi). The PCR band generated by primers specific to the predicted recombinant (29F-19R, forward primer for HAdV-D29 HVL1 and reverse primer for HAdV-D19 HVL2) was greater in K-12 lysate-pretreated cells. Control PCR with primers that do not distinguish recombinants from parent viruses is shown below (PentonF-R). (D) Quantitative PCR under the same treatment conditions in panel C shows relative homologous recombination levels, as normalized to PBS-treated cells (*, *P* < 0.05 by ANOVA). Each experiment was performed in triplicate and repeated three times. Error bars represent standard deviations of the means.

**FIG 4:**
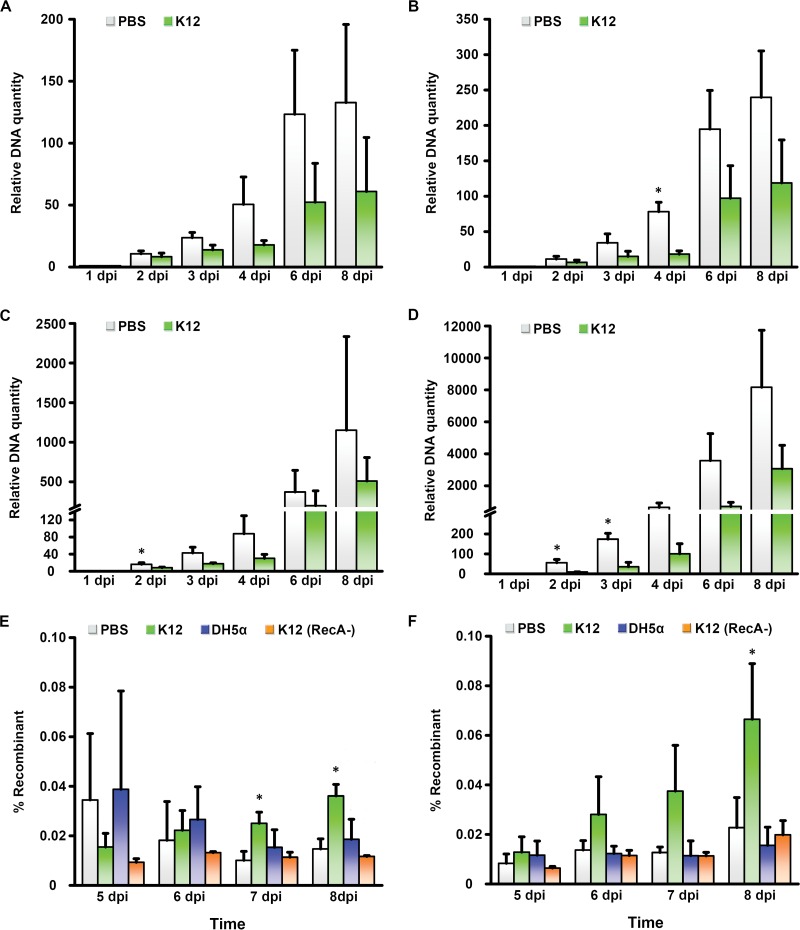
Relative viral replication and recombination in the presence of bacterial protein. Using primers specific to hexon sequence in HAdV-D19 (A and C) and HAdV-D29 (B and D), quantitative PCR was used to quantify total viral DNA from 1 to 8 days postinfection in PBS-treated versus *E*. *coli* K-12 lysate-treated C2BBe1 cells (A and B) and A549 cells (C and D). DNA quantity is graphed relative to the levels at 1 day postinfection. (E and F) C2BBe1 (E) and A549 (F) cells pretreated with PBS, K-12 lysate, DH5α lysate, or K-12 lysate depleted of RecA were coinfected with HAdV-D19 and HAdV-D29 and subjected to quantitative PCR at 5 to 8 days postinfection, with primers chosen to amplify only the HVL2 recombinant. Values that are significantly different (*P* < 0.05) by ANOVA are indicated by an asterisk. Each experiment was performed in triplicate and repeated three times. Error bars represent standard deviations of the means.

The human gut microbiota includes trillions of bacteria and countless bacteriophage (47). Their evolution is driven in part by Rec protein-mediated homologous recombination, specifically the unwinding of dsDNA by the RecBCD complex and upon recognition of Chi, the loading of RecA onto the ssDNA (48). We quantified homologous recombination between Chi_AD_ constructs in the presence of an endotoxin-free lysate of E. coli strain K-12 in comparison to a lysate from the K-12 RecA mutant strain DH5α. By trypan blue exclusion, bacterial cell lysates induced no cell toxicity (data not shown). As predicted, the K-12 lysate promoted more homologous recombination between constructs than the DH5α lysate (Fig. 3A). The presence of RecA in lysates from each strain was confirmed by Western blotting. To determine whether the effect was specific to RecA, we also tested K-12 lysates from which we depleted RecA protein with immunomagnetic beads and found that RecA-depleted K-12 lysate induced less recombination than lysate with mock depletion (Fig. 3B). We next examined the effects of lysates from K-12, DH5α, and RecA-depleted K-12 on penton base HVL2 recombination between two wild-type viruses with conserved Chi_AD_, HAdV-D19 and HAdV-D29, chosen because of sufficient sequence disparity in HVL1 and HVL2 to permit PCR discrimination of recombinants. We coinfected both viruses in the intestinal adenocarcinoma cell line Caco-2 (clone C2BBe1) and used conventional and quantitative PCR (qPCR) (Fig. 3C and D, respectively) to identify recombination of penton base HVL2 between viruses. *E*. *coli* K-12 lysate promoted recombination relative to phosphate-buffered saline (PBS), and to a greater degree than either *E*. *coli* DH5α or RecA-depleted K-12, with the effect statistically significant by 7 days postinfection. The same results were evident in A549 cells, a lung carcinoma cell line (Fig. S3). Because an increase in viral replication due to bacterial lysate could have accounted for the apparent increase in recombined viral DNA (49), we also infected each cell type in the presence of K-12 lysate or PBS control and performed qPCR with primers specific to sequence conserved between the hexon genes of both viruses (Table S1). Relative to treatment with PBS, K-12 lysate appeared to reduce but not prevent viral replication through 8 days postinfection in either cell line (Fig. 4A to D). Upon coinfection and subsequent assay by qPCR, the recombinant accounted for less than 0.1% of total viral DNA in either C2BBe1 (Fig. 4E) or A549 cells (Fig. 4F). The relative increase in the ratio of recombinant viral DNA to total viral DNA in the presence of K-12 lysate was maintained when total viral replication was taken into account. Therefore, the absolute number of viral recombinants increased in the presence of bacterial lysates despite a general inhibition of viral replication.

10.1128/mSphere.00105-18.3 FIG S3  Promotion of homologous recombination by bacterial lysate in the A549 lung carcinoma cell line. (A) Conventional PCR performed on A549 cells pretreated with either PBS, *E*. *coli* K-12 lysate, *E*. *coli* DH5α lysate, or RecA-depleted K-12 lysate, and coinfected with HAdV-D19 and HAdV-D29, from 3 to 8 days postinfection. The PCR band generated by primers specific to the predicted recombinant (29F-19R is the forward primer for HAdV-D29 HVL1 and the reverse primer for HAdV-D19 HVL2) was greater in K-12 lysate-pretreated cells. Control PCR with primers that do not distinguish recombinants from parent viruses is shown below (PentonF-R). (B) Quantitative real-time PCR under the same treatment conditions shows relative homologous recombination levels normalized to the levels for PBS-treated cells (*, *P* < 0.05 by ANOVA). Each experiment was performed in triplicate and repeated three times. Error bars represent standard deviations of the means. Download FIG S3, PDF file, 0.9 MB. Copyright © 2018 Lee et al. 2018 Lee et al. This content is distributed under the terms of the Creative Commons Attribution 4.0 International license.

**FIG 5:**
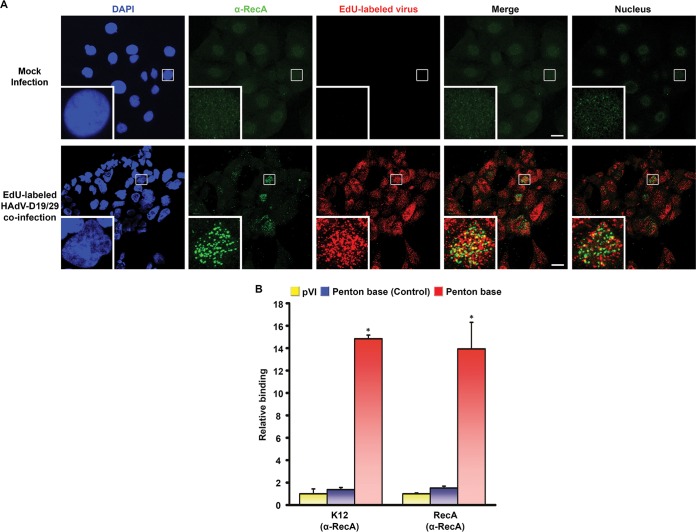
Binding of RecA to Chi_AD_ in intestinal epithelial cells. (A) Confocal microscopy in C2BBe1 cells pretreated with *E*. *coli* K-12 lysate for 24 h and then either mock infected or coinfected with EdU-labeled (red) HAdV-D19 and HAdV-D29. Samples were fixed at 12 h postinfection and stained with DAPI (blue) and anti-RecA (green). Stacked images without blue color are shown in the Merge panels (bars, 25 µm). To reduce any artifact of perinuclear localization, a single image centered on the nucleus in the inset with one image on either side is also shown in the Nucleus panels. Colocalization of viral DNA and RecA is suggested by the yellow color. The small white boxes in the micrographs show the locations of the insets. Original magnification, ×63. (B) ChIP analysis performed on C2BBe1 cells pretreated with either *E*. *coli* K-12 lysate or recombinant RecA prior to coinfection with HAdV-D19 and HAdV-D29. Binding of RecA to Chi_AD_ was compared to IgG (not shown) and randomly chosen regions in protein VI (pVI) and penton base (Control). Binding affinities were normalized to pVI binding of RecA protein. Each experiment was repeated three times. Error bars represent standard deviations of the means. For each experiment, *, *P* < 0.05 by ANOVA.

Adenovirus-infected cells at the time of viral replication have lost the capacity for cellular biosynthesis, and these cells are in the early phases of virus-induced cell death with reduced integrity to their cellular membranes (50). In addition, HAdV-D37 was previously shown to release the ectodomain of MUC16 from ocular surface cells, suggesting that adenoviruses may have intrinsic means of reducing overall mucosal barrier function (51). We next performed confocal microscopy to determine whether RecA would colocalize with viral DNA in infection of C2BBe1 cells (Fig. 5A). Coinfection with 5-ethynyl-2′-deoxyuridine (EdU)-labeled HAdV-D19 and HAdV-D29 was performed in the presence of E. coli K-12 lysate, and the cells were imaged at 12 h postinfection. RecA protein was identified only in the nuclei of infected cells, where it colocalized with viral DNA. Confocal microscopy for the presence of RecB, RecC, and RecD similarly showed nuclear colocalization of those proteins with viral DNA (Fig. S4). Healthy, uninfected cells excluded Rec proteins. We next performed chromatin immunoprecipitation (ChIP) 6 days after coinfection of C2BBe1 cells pretreated with K-12 lysate. Binding of the IgG control was extremely low (data not shown), so we compared RecA binding of HVL2 Chi_AD_ with binding of RecA to conserved genomic regions without Chi_AD_: one in protein VI and another at the conserved 3′ end of the penton base gene. RecA binding to penton base sequence containing Chi_AD_ was ~14-fold greater than to control regions of HAdV DNA (Fig. 5B). There are noncanonical pathways of RecA loading that function in the absence of RecBCD in phage (52). To determine whether RecBCD or other bacterial proteins are required for a putative interaction between RecA and Chi_AD_, we repeated ChIP with recombinant RecA instead of E. coli lysate. The ratios of binding to Chi_AD_ relative to other regions of the genome were similar (Fig. 5B). Relative to IgG, binding with K-12 lysate was twice that observed with recombinant RecA (data not shown). These data show specificity of RecA binding to Chi_AD_ and suggest that RecBCD dispensably improves binding.

10.1128/mSphere.00105-18.4 FIG S4  Bacterial Rec protein colocalization with adenoviral DNA in cell nuclei. Confocal microscopy of C2BBe1 cells pretreated with *E*. *coli* K-12 lysate for 24 h and then mock infected or coinfected with EdU-labeled (red) HAdV-D19 and HAdV-D29 for 12 h. Samples were fixed at 12 h postinfection and stained with DAPI (blue) and antibody against RecB (A), RecC (B), or RecD (C) (green). Stacked images without blue color are shown in the Merge panels (bars, 25 µm.). To reduce any artifact of perinuclear localization, a single image centered on the nucleus in the inset with one image on either side is also shown in the Nucleus panels. Colocalization of viral DNA and each Rec protein is suggested by the yellow color. The small white boxes in the micrographs show the locations of the insets. Original magnification, ×63. Download FIG S4, PDF file, 1.9 MB. Copyright © 2018 Lee et al. 2018 Lee et al. This content is distributed under the terms of the Creative Commons Attribution 4.0 International license.

## DISCUSSION

HAdV infects mucosal sites to cause a myriad of human diseases, and it can be lethal in the immunocompromised host (53). How adenoviruses evolve is therefore of considerable significance, as new types can be associated with enhanced virulence and pathogenicity. Single nucleotide substitutions in HAdV genomes are relatively slow to accrue, with nucleotide-specific stability seen over decades (13, 54). In contrast, homologous recombination among circulating HAdV is widely recognized as the principal means of their evolution (49, 55–62), and recombinational evolution of HAdV is now codified in GenBank/NCBI criteria for typing newly emerging or historically known but previously uncharacterized HAdV (63, 64). In HAdV-D, specific homologous recombination patterns predominate (14, 19, 41–43, 65–68). For example, homologous recombination involving the hypervariable region corresponding to penton base HVL2 is particularly widespread (19), seen in almost every HAdV-D analyzed thus far. The emergent HAdV-D53, now a common cause of severe epidemic keratoconjunctivitis (69), expresses hexon epitopes of the nonpathogenic HAdV-D22, but the penton base of HAdV-D37, a previously characterized and highly virulent eye pathogen (43). Another eye pathogen, HAdV-D64, expresses the hexon epitopes of the nonpathogenic HAdV-D19 on a genome “chassis” of HAdV-D37 with its penton base gene contributed by HAdV-D22 (68). Homologous recombination is not restricted to HAdV-D. In HAdV-B55, homologous recombination of the hexon gene hypervariable regions, prime determinants of type-specific humoral immune responses, permitted the escape of a serious respiratory pathogen from immune pressure (70–74).

Recombination between adenoviruses requires at least two homologous adenoviral sequences, one an intact dsDNA and the other an ssDNA, as would be present in cells undergoing viral DNA replication during coinfection (59, 60). Coinfection with two or more HAdV types occurs commonly (12, 75, 76) and is tolerated by the host because host immunity is mostly type specific (77). HAdV has been shown to cause persistent infections of healthy persons (37) within secondary lymphoid tissues, including those at Waldeyer’s ring (tonsils and adenoids), the gastrointestinal tract (4, 78–84), and even the ocular surface (85). Viral persistence in infected tissues increases the likelihood of coinfection with two or more adenoviruses. However, host factors that promote recombination between HAdV genomes *in vivo* are unknown. Homologous recombination was first identified by Lederberg and Tatum in 1946 (86) as a means to ensure the viability of phage and bacteria under host and environmental selection pressures and as a repair mechanism for dsDNA and ssDNA breaks during genome replication (87–89). Homologous recombination between bacterial genomes is driven by the presence of the ubiquitously expressed, bacterial RecA (38-kDa) protein, which is loaded onto bacterial and phage DNA by the heterotrimer RecBCD or alternate Rec proteins. RecA-mediated strand exchange (branch migration) occurs upon strict base pairing between adjacent DNA molecules (90), and the recombination event can then extend to include thousands of subsequent base pairs. Experimental null mutations in E. coli RecA diminished bacterial recombination as much as 100,000-fold, while mutations in the other Rec proteins had substantially lower effects (91), consistent with known redundancy in the RecA loading function of RecBCD, but not in the recombination function of RecA. Although we showed a statistically significant increase in viral recombination in the presence of bacterial lysate, with reduced effect upon RecA depletion, the impact on generation of new recombinant viral DNA was modest in comparison to the known effect of RecA on bacteria and phage. If HAdV-Ds utilize bacterial recombination machinery *in vivo*, they likely do so with considerably lower efficiency than for bacteria and phage.

Chi sequences are typically 8 nucleotides long with a thymidine (T) at the third position, but they differ in bacterial genera. Chi sequences appear more frequently than would be expected by chance alone, suggesting their positive selection to facilitate DNA repair and recombinational evolution (25). In bacteria and phage, the specific and highly conserved nucleotide sequence for Chi is critical to its function, while in our experiments, Chi_AD_ sequence specificity appeared considerably less stringent. Strictly speaking, confirmation of a canonical Chi-like effect of Chi_AD_ will require further experimental study (92). However, Chi-like sequences have been identified in human immunodeficiency virus (93) and in human immunoglobulin and ABO genes (94, 95) and implicated in translocation-associated human malignancies (96–99). We identified Chi-like sequences in HAdV-D genomes (Chi_AD_) at a known recombination hot spot, just 5′ to the sequence encoding penton base HVL2, the latter critical to viral internalization and a presumed pathogenesis determinant. We showed in DNA constructs that Chi_AD_ sequence and directionality impact recombination, although not with the same degree of sequence specificity as Chi in bacteria. Regardless, HAdV-D coinfection in the presence of E. coli lysate increased recombination, not seen with a RecA mutant strain or with RecA depletion, and RecA entered HAdV-infected cell nuclei where it localized with viral DNA. Therefore, although RecA-mediated adenoviral recombination appears to be less efficient and less sequence specific than in bacteria and phage, it nevertheless appears that adenoviruses can benefit from the recombination machinery of resident bacterial flora. While adenoviruses may exploit the recombination machinery of local bacterial flora, the efficiency of replication by the recombinant virus and subsequent selection pressure by the host would determine whether a new recombinant survives and is transmitted. In other words, postrecombination selection must also play a role in the emergence of any new viruses. Regardless, our work is consistent with the idea that human, bacterial, and viral genomes in the gut may utilize common recombination machinery to foster microbial and viral diversity. It is also possible that adenoviral recombination occurs during infection of other mucosal sites. For instance, while the presence of a stable conjunctival microbiome is a matter of some conjecture (100, 101), it is clear that bacterial superinfection coincident to adenovirus infection can occur in epidemic keratoconjunctivitis (102, 103), which could conceivably promote adenoviral recombination at the ocular surface.

Secondary structure is also known to impact recombination between two homologous nucleic acid molecules. Our analysis showed recombination in one construct (SY) not predicted to undergo recombination, but the secondary structure and location of Chi_AD_ within that structure were highly similar to those of the parent Chi_AD_. Imputed secondary structures were also found to be highly similar within but not between penton base HVL2 proteotypes. In RNA viruses, stem-loop secondary structures were shown to be particularly critical to recombination between two homologous ssRNA (104–106). Adjacent regions that were 5′ GC-rich and 3′ AU-rich (39, 40) were considerably more likely to recombine in brome mosaic virus (107), particularly when the region of relatively greater GC content is followed by a greater AU-rich content of equal length with preference for a GC/AU transition of 30, 45, or 60 nucleotides. The AU-rich regions formed hairpin loops thought to induce a pause of the RNA replicase, followed by template switching of the replicase (40, 108), i.e., polymerase jumping, when loop-to-loop complementarity was also present (106). Polymerase hesitation due to secondary loop structures was also shown to promote homologous recombination between retroviruses (109, 110), poliovirus (111), norovirus (112), and coronavirus (113), suggesting a common mechanism.

A relationship between a virus and local bacterial flora could be detrimental to one or both, beneficial to one or both, or even obligate, with the outcome likely to shift over time due to the dynamics of constantly changing host factors and environmental influences. Commensalism characterizes a relationship between two disparate organisms when one organism benefits while the other is unaffected. Certain RNA viruses benefit by hijacking bacterial surface glycans in the gut to achieve host cell entry (29). We propose a novel example of viral commensalism in which Chi_AD_ sequences in the HAdV-D genome are bound within infected cells by bacterial RecA, present because of local bacterial flora, to facilitate homologous recombination between viruses. Free Rec proteins may be present in gastrointestinal secretions because of bacterial senescence and loss of structural competency, competition and killing by other bacterial species vying for the same mucosal niche, bacteriophage-mediated bacterial cell lysis, host inflammatory cells and factors induced by adenovirus infection that can lyse bacterial cells, and/or the presence of endogenous antimicrobial peptides at the mucosal surfaces (114–119). However, to characterize the relationship as commensal may be an oversimplification, as the interactions between adenoviruses, bacterial flora, and the host are certain to be multifaceted. For example, α-defensins, peptides generated by the host in response to microbes present in the gastrointestinal tract, limited adenovirus uncoating within endosomes (120, 121) and potentiated neutralizing antibody responses to infection (122), but also enhanced viral entry into host cells (123). Our work is another example of complexity in the dynamic evolution of virus-host interactions, including the host’s microbiota.

## MATERIALS AND METHODS

### Cells, viruses, and bacteria.

The 293A cell line was obtained from Thermo Fisher Scientific (Waltham, MA) (R70507), and the A549 and Caco-2 (C2BBe1 clone) cell lines were obtained from ATCC (Manassas, VA) (ATCC CCL-185 and ATCC CRL-2102). Cell lines were tested for and verified as *Mycoplasma* negative. Escherichia coli strain K-12 (ATCC 10798) was purchased from ATCC. E. coli strain DH5α was a gift from Michael Gilmore at Massachusetts Eye and Ear Infirmary, Harvard Medical School. HAdV-D19 (ATCC VR-1096) and HAdV-D29 (ATCC VR-1107) were purchased from ATCC and verified by molecular typing of the major capsid genes. Viruses were purified using the cesium chloride gradient method, verified as endotoxin negative, and the titers of the virus were determined by the tissue culture infectious dose method.

### GC/AT transitions and identification of Chi-like motifs.

HAdV-D gene segments of interest in 38 viruses of HAdV-D were organized by maximum likelihood trees, constructed using MEGA5 (http://www.megasoftware.net/), and segregated into proteotypes by 10% difference in amino acid content and confirmed by simple inspection. Crossover hot spot instigator (Chi) sequences inside GC/AT transition zones were identified by searching for 5′-NNTNNTNN-3′ in which N could be any nucleotide. The same 38 viruses in HAdV-D were studied individually with the Recombination Site Pattern Finder (14). A threshold of 10% difference in GC content was applied to identify regions of 30-, 45-, or 60-nucleotide transition from GC rich to AT rich, with a sliding 15-nucleotide window. All GC/AT transition zones for 38 viruses were combined by matching nucleotide positions.

### Cloning.

The pcDNA3.1-hygromycin vector was obtained from Thermo Fisher Scientific, and the hygromycin resistance gene replaced with one for neomycin from pcDNA3.1-myc-his-A(+) (Thermo Fisher Scientific). The cytomegalovirus (CMV) and T7 promoters (pCVMT7) of both vectors were removed using NruI-HF and NheI-HF, restriction enzymes from New England BioLabs (NEB, Ipswich, MA), and an adaptor for Sanger sequencing (see Table S1 in the supplemental material) synthesized at Integrated DNA Technologies (IDT) was cloned into the same region using T4 DNA ligase (NEB) to generate pcDNA3.1-hygromycine/neomycin-NoCMVT7. Using Q5 Hot Start high-fidelity DNA polymerase (NEB), CMVT7 promoter and green fluorescent protein (GFP) genes without start codon were amplified from pcDNA3.1-hygromycin and pEGFP-N1 (EGFP stands for enhanced GFP) (TaKaRa, Mountain View, CA), respectively. The CMVT7 promoter was inserted into the penton base gene of HAdV-D64 between nucleotides 14414 and 14415, just after the GC/AT transition zone marking the transition from conserved sequence to HVL2 (D64CMV). The GFP gene without a start codon was inserted into the penton base gene of HAdV-D22 between nucleotides 15073 and 15074 (D22GFP). D64CMV and D22GFP were then cloned into pcDNA3.1-hygromycin-NoCMVT7 and pcDNA3.1-neomycin-NoCMVT7 vectors, respectively. Chi_AD_ mutants were created on both pcDNA3.1-hygromycin-NoCMVT7-D64CMV and pcDNA3.1-neomycin-NoCMVT7-D22GFP vectors using overlap extension PCR. All constructs were verified by Sanger sequencing (Ocular Genomics Institute, Massachusetts Eye and Ear, Boston, MA).

### Transfection and measurement of GFP signal.

293A cells were seeded on black 96-well cell culture plates (Greiner Bio-One, Monroe, NC) in Dulbecco’s modified Eagle medium (DMEM), with 10% fetal bovine serum (FBS) and 1% penicillin-streptomycin (Thermo Fisher Scientific). After incubation at 37°C in 5% CO_2_ for 24 h, 100 ng of each plasmid per well was transfected using Lipofectamine 3000 (Thermo Fisher Scientific) according to the manufacturer’s instructions. At 2 days posttransfection, GFP expression level in each well was measured on a SpectraMax M2 microplate reader (Molecular Devices, Sunnyvale, CA) and visualized on a Leica SP5 confocal system (Leica Microsystems, Buffalo Grove, IL) with 40× magnification. For comparison of GFP expression levels in the presence of bacterial lysates, 293A cells were treated with 1 µg/well of either *E*. *coli* K-12, DH5α, or RecA-depleted (RecA-) lysate for 24 h prior to transfection.

### PCR and quantitative PCR.

PCR primers were synthesized by Integrated DNA Technologies (IDT) (Coralville, IA), and these primers are shown in Table S1. Viral DNA was isolated using GeneJET viral DNA/RNA purification kit (Thermo Fisher Scientific) per the manufacturer’s instructions. DNA was quantified and quality checked on a NanoDrop 2000C spectrophotometer (Thermo Fisher Scientific). PCR was performed on a PTC-200 thermal cycler (Bio-Rad, Hercules, CA) with 1 ng of DNA, 12.5 µl of GoTaq G2 hot start green master mix (Promega, Madison, WI), and 30 ng of forward and reverse primer with each primers in a total volume of 25 µl. PCR was performed as follows: 95°C for 2 min; 35 cycles, with 1 cycle consisting of 95°C for 30 s, 60°C for 1 min, 72°C for 35 s, and 72°C for 5 min. Quantitative PCR (qPCR) was performed on a QuantStudio 3 real-time PCR system (Thermo Fisher Scientific) with 1 ng of DNA, 10 µl of Fast Sybr green master mix (Thermo Fisher Scientific), and 20 ng of forward and reverse primer with each primer in a total volume of 20 µl. qPCR was performed as follows: 95°C for 20 s; 40 cycles, with 1 cycle consisting of 95°C for 1 s and 60°C for 20 s with data collection and melting curve analysis.

### Secondary structure modeling.

The mFold Web Server’s DNA folding form was used for DNA secondary structure analysis and free energy measurements, without application of constraints, and set for a linear DNA sequence with a folding temperature of 37°C. For default values, ionic conditions were specified at 1 M sodium and no magnesium, maximum distance between paired bases as unlimited, percent suboptimality number fixed at 5, structure rotation angle set at auto, and regularization angle at 0°.

### E. coli lysate preparation and treatment.

*E*. *coli* K-12 and DH5α were each inoculated into Luria broth (LB), incubated at 37°C for 16 h in a shaking incubator at 225 rpm, then collected by centrifugation in 50-ml conical tubes at 5,000 rpm for 10 min, and resuspended in 10 ml of phosphate-buffered saline (PBS), and aliquots of the bacterial solutions were added to Lysing Matrix B tubes (MP Biomedicals, Santa Ana, CA). Lysis was performed using a FastPrep-24 5G instrument (MP Biomedicals) at 6,000 rpm for six 45-s pulses. Endotoxin was extracted from bacterial lysates with Pierce High Capacity Endotoxin Removal spin columns (Thermo Fisher Scientific). Protein concentrations were measured by Pierce BCA protein assay kit (Thermo Fisher Scientific). Lysate was added to each well of six-well cell cultures at a final concentration of 50-µg bacterial protein/ml of culture medium, i.e., 100 µg/tissue culture well. For PBS control, an equivalent volume of PBS was added to tissue culture medium per well. The presence of endotoxin in bacterial lysates was ruled out using the ToxinSensor chromogenic LAL (*Limulus* amebocyte lysate) endotoxin assay kit (GenScript, Piscataway, NJ); the final endotoxin levels in cell culture medium after the addition of bacterial lysates were below the detection limit of 0.005 endotoxin unit (EU)/ml.

RecA depletion from the *E*. *coli* K-12 strain was performed with magnetic beads. After *E*. *coli* was vortexed for 1 min, 50-µl aliquots of Dynabeads M-280 sheep anti-rabbit IgG (Thermo Fisher Scientific) were added to 1.5-ml Eppendorf tubes and suspended in 1 ml of washing buffer consisting of 0.1% bovine serum albumin (BSA) in magnesium- and calcium-free PBS. The tubes were placed in a MagJET magnetic separation rack (Thermo Fisher Scientific) for 2 min, and then the wash buffer was removed. Five hundred microliters of fresh wash buffer and 5 µg of anti-RecA antibody (catalog no. ab63797; Abcam, Cambridge, MA) were added to the beads, and after gentle mixing, the tubes were incubated at 4°C for 2 h in rotation. Control lysates (without RecA depletion) were treated with rabbit IgG isotype control (catalog no. Ab37415; Abcam). The tubes were then placed in the magnetic separation rack for 2 min, and the supernatant was removed. The beads were washed with 1 ml of washing buffer three times. Five hundred micrograms of *E*. *coli* K-12 lysate in 500 µl was added and incubated at 4°C for 2 h in rotation. After the tubes were placed in the magnetic separation rack for 2 min, the supernatant was transferred into new tubes. Fifty microliters of elution buffer (0.1 M citrate [pH 2.3]) was added to the beads and boiled for 5 min. The tubes were placed in the magnetic separation rack for 2 min, and the buffer was transferred into new tubes. The protein concentration was measured by using the Pierce BCA protein assay kit as described above, and 20-µg portions were used for Western blotting with anti-RecA (catalog no. MD-03-3; MBL International, Woburn, MA) and anti-β-galactosidase (catalog no. ab616; Abcam).

Bacterial lysate toxicity was assessed by trypan blue exclusion; treatment of cell cultures with 50 µg bacterial lysate/ml of tissue culture medium for 1 week showed no cellular toxicity. C2BBe1 and A549 cells were seeded in six-well plates (Corning, Corning, NY). After 24-h incubation, 100 µg in 50 µl for each bacterial lysate was added to 2 ml DMEM with 1% insulin-transferrin-selenium in each well for 24 h, followed by coinfection with HAdV-D19 and HAdV-D29, each at a multiplicity of infection (MOI) of 0.001. One hundred eighty microliters of supernatant was collected from 3 days postinfection (dpi) to 8 dpi. Two units of DNase I (NEB) was treated with 20 µl of 10× DNase I buffer at 37°C for 1 h, and then 2 µl of 0.5 M EDTA (pH 8.0) was added and incubated at 75°C for 10 min to inactivate DNase I, prior to viral DNA isolation and PCR.

### Confocal microscopy.

C2BBe1 cells were cultured on four-well Nunc Lab-Tek chamber slides (Thermo Fisher Scientific) for 24 h. Bacterial lysate was added to each well as described above for another 24 h prior to viral infection. 5-Ethynyl-2′-deoxyuridine (EdU)-labeled HAdV-D19 and HAdV-D29 were prepared using Click-iT Plus EdU Alexa Fluor 555 imaging kit (Thermo Fisher Scientific) and added to each well at an MOI of ~25 (for each virus) and incubated at 37°C in an incubator with 5% CO_2 _for 12 h. The cells were washed three times with PBS, fixed for 10 min in 300 µl of 4% paraformaldehyde, washed three times in PBS containing 2% BSA, treated with 300 µl of permeabilization buffer (0.1% Triton X-100 in washing buffer) for 10 min, and washed again. The Click-iT Plus reaction cocktail was added for 30 min at room temperature, protected from light. After the cells were washed three times, 300 µl of PBS containing 2% BSA was added for 30 min for blocking. Primary antibodies were prepared as follows: 1:1,000 dilution for anti-RecA (MBL International) in washing buffer, 1:2,000 dilution for anti-RecB, anti-RecC, and anti-RecD (gifts of Gerry Smith at Fred Hutchinson Cancer Research Center, Seattle, WA). Three hundred microliters of each primary antibody was incubated with the cells for 1 h at room temperature, protected from light. After the cells were washed three times, 300 µl of 1:5,000 dilution in washing buffer of secondary antibody was added to goat anti-mouse IgG-Alexa Fluor 488 conjugate (Thermo Fisher Scientific) for anti-RecA and to goat anti-rabbit IgG-Alexa Fluor 488 conjugate (Thermo Fisher Scientific) for anti-RecB, anti-RecC, and anti-RecD and incubated for 45 min at room temperature. After three washes each in washing buffer and then in PBS, the cells were mounted with Vectashield antifade mounting medium containing 4′,6′-diamidino-2-phenylindole (DAPI) (Vector Laboratories, Burlingame, CA). Photomicrographs were obtained on the Leica SP5 confocal system with 63× magnification.

### Chromatin immunoprecipitation.

C2BBe1 cells were seeded on 100-mm tissue culture dishes (Greiner Bio-One) for 24 h, and 500 µg of *E*. *coli* K-12 lysate was added for an additional 24 h. HAdV-D19 and HAdV-D29 were added to the cells at an MOI of 0.001, with bacterial lysate at the same concentration. At 6 dpi, cells were collected by scraping into PBS, centrifuged, and washed twice in PBS. DNA was extracted using a Pierce magnetic ChIP (chromatin immunoprecipitation) kit (Thermo Fisher Scientific) per the manufacturer’s instructions, sheared with a Q700 sonicator (Qsonica, Newtown, CT) and then treated with micrococcal nuclease (MNase). The sheared DNA was run on an agarose gel to confirm DNA fragments ranging between 200 and 1000 bp in length. ChIP assays were performed with anti-RecA antibody (Abcam), using the same Pierce kit with qPCR primers as listed in Table S1.

### Statistical analysis.

All experiments were performed at least three times. Data were analyzed by either Student’s *t* test for pairwise comparisons or by analysis of variance (ANOVA) with preplanned comparisons, using SAS (Cary, NC). Significance was set *a priori* at *P* < 0.05.
