# Supplementary figures and images for: Bacterial RecA Protein Promotes Adenoviral Recombination during In Vitro Infection
Source: mSphere. 2018 Jun 20;3(3):e00105-18. doi: 10.1128/mSphere.00105-18 (PMC6010623; doi:10.1128/mSphere.00105-18)

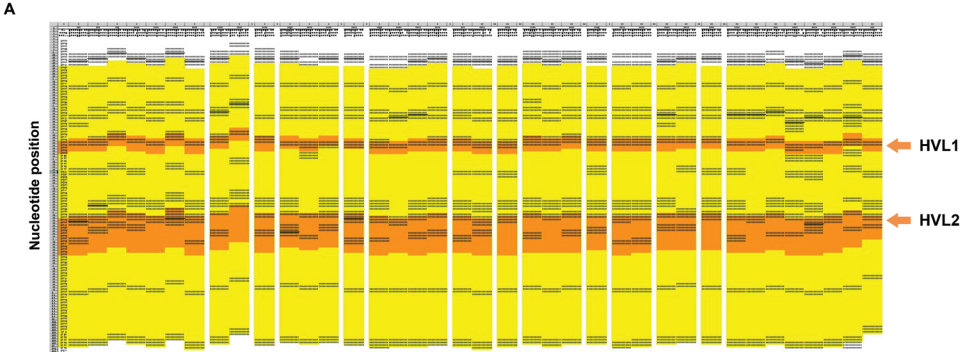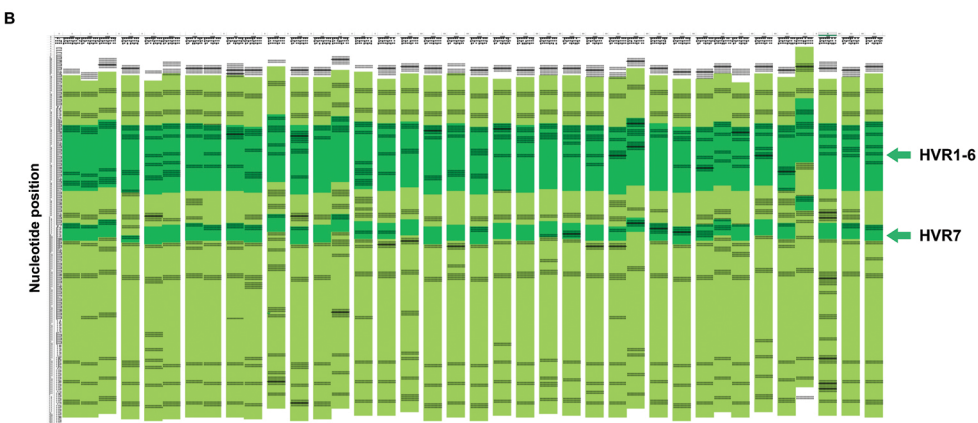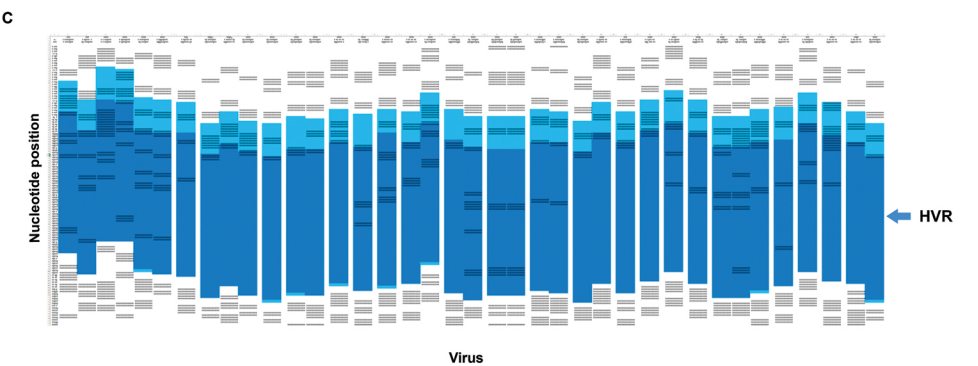

Supplement: FIG S1 [file sph003182573sf1.pdf]

D42

D59

D22

D64

D45

D42

D59

D22

D64

D45

D38

D24

D27

D48

D24

D27

D48

D58

D8

D49

D32

D44

D58

D8

D49

D32

D44

D30

D51

D19

D23

D33

D30

D51

D19

D23

D33

D43

D29

D36

D25

D46

D43

D29

D36

D25

D46

D47

D26

D56

D10

D39

D9

D26

D56

D10

D39

D9

D54

D13

D37

D53

D60

D69

D20

D28

D13

D37

D53

D60

D69

D20

D28

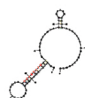

D38

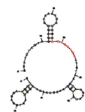

D47

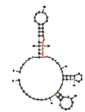

D54

Supplement: FIG S2 [file sph003182573sf2.pdf]

**A**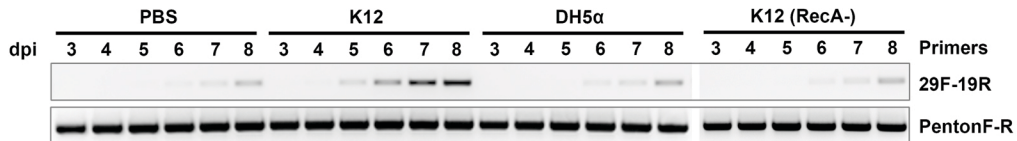**B**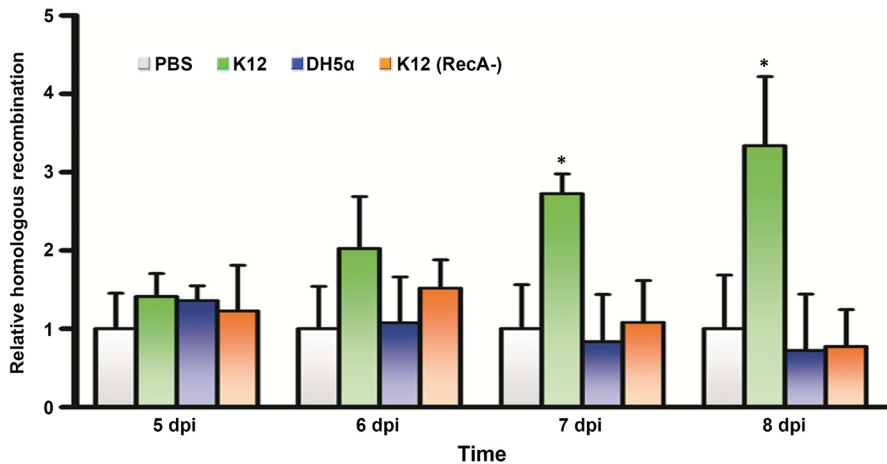

Supplement: FIG S3 [file sph003182573sf3.pdf]
