# Supplementary material for: Bacterial RecA Protein Promotes Adenoviral Recombination during In Vitro Infection
Source: mSphere. 2018 Jun 20;3(3):e00105-18. doi: 10.1128/mSphere.00105-18 (PMC6010623; doi:10.1128/mSphere.00105-18)
Supplement: TABLE S1 [file sph003182573st1.docx]

**Supplemental Table.** PCR primers and nucleotide sequences.

| **Primers** | **Sequences** | **Positions** | |
| --- | --- | --- | --- |
| **PCR and qPCR** |  |  |  |
|  |  | **D19** | **D29** |
| D29-HVL1-F | ACC CAC AGG GGG TAG AAG CAA CAG ATT TAA GC |  | 13941-13972 |
| D19-HVL2-R | GGG CCG CTT TCT GTT TTG CAT CTT CAA TTT CT | 14405-14374 |  |
| Penton-F | TGA TGC CAG GGG TCT ACA CCT ACG AGG CCT | 14160-14189 | 14178-14207 |
| Penton-R | TAG AGC GGA AGG TGA CGG GGT CTT GCA TGA | 14702-14673 | 14729-14700 |
| Penton-ChIP-F | GAA GCA ACC TTT CCA AGA GGG CTT CAG | 14272-14298 | 14290-14316 |
| Penton-ChIP-R | GTA TCC AGG AGA GCG GGG ATG TTA CC | 14349-14324 | 14367-14342 |
| Penton-ChIP-Con-F | CAC AGA TCA CGG GAC GCT AC | 14911-14930 | 14938-14957 |
| Penton-ChIP-Con-R | TTA GAA GGT GCG ACT GGA AAG C | 15058-15037 | 15085-15064 |
| pVI-ChIP-F | ATG AAA CCT GCC ACC CTT GAC | 17506-17526 | 17536-17556 |
| pVI-ChIP-R | CGA TAC TGT GCA GCG TGC TCT | 17672-17658 | 17689-17669 |
| D19-Hexon-F | CCC TTC CAG CAC AAC AAC TCG | 20228-20248 |  |
| D19-Hexon-R | ACC CTG TCG CAG AGG AAC TTT | 20376-20356 |  |
| D29-Hexon-F | GGC AAA CAA GAC CCG ATA TAT GCT AAT AAG CTG TAT C |  | 18319-18355 |
| D29-Hexon-R | CAA AAT CTT CTT GAC TTT TTT CAG GGT CTC TTA GTT TTG |  | 18494-18532 |
| GAPDH-F | TGG GCT ACA CTG AGC ACC AG |  |  |
| GAPDH-R | ACC ACC CTG TTG CTG TAG CC |  |  |
|  |  |  |  |
|  |  |  |  |
| **Cloning** |  |  |  |
|  |  | **D22** | **D64** |
| Adaptor | TCG CGA GTG GTT GGC TAC GTA TAC TCC GGT GTA CGG GCC AGA TAT ACG CGT TGA CAA GCT AGC |  |  |
| D22/D64-Penton-HindIII-F | CCC AAG CTT ACC ATG AGG CGT GCG GTG GTG TCT TCC TCT | 13523-13549 | 13537-13562 |
| D64-Penton-XhoI-R | CCG CTC GAG TTA GAA GGT GCG ACT GGA AAG CAC GCG |  | 15081-15055 |
| D22-Penton-NoStop-BamHI-R | CGC GGA TCC GAA GGT GCG ACT GGA AAG AAC GCG | 15073-15050 |  |
| D64-Penton-CMV-3SPCR-1R | ATT GAT TAC TAT TAA TAA CTA GTC AAT AAT CAA TGT TCT AGC TTC TTC TTG CTT TCC AGG |  | 14414-14391 |
| CMVT7-F | ACA TTG ATT ATT GAC TAG TTA TTA ATA GTA ATC AAT TAC G |  |  |
| CMVT7-R | CCT ATA GTG AGT CGT ATT AAT TTC GAT AAG CCA G |  |  |
| D64P-CMV-3SPCR-3F | CTG GCT TAT CGA AAT TAA TAC GAC TCA CTA TAG GGG AGA ATG CCG CTA AGG CTA ATG GTC |  | 14414-14391 |
| EGFP-BamHI-NoStart-F | CGC GGA TCC GTG AGC AAG GGC GAG GAG CTG TTC ACC |  |  |
| EGFP-XhoI-R | CCG CTC GAG TTA CTT GTA CAG CTC GTC CAT GCC GAG AGT G |  |  |
| NonChi-SA-F | GGG CAA CAT TCC CGC GTT ATT AAA TGT GAC CAA GTA CCT G | 14341-14380 | 14355-14394 |
| NonChi-SA-R | CAG GTA CTT GGT CAC ATT TAA TAA CGC GGG AAT GTT GCC C |  |  |
| NonChi-RE-F | GGG CAA CAT TCC CGC AGT CTT CAA TGT GAC CAA GTA CCT G |  |  |
| NonChi-RE-R | CAG GTA CTT GGT CAC ATT GAA GAC TGC GGG AAT GTT GCC C |  |  |
| NonChi-AS-F | GGG CAA CAT TCC CGC TGA AGA CTA TGT GAC CAA GTA CCT G |  |  |
| NonChi-AS-R | CAG GTA CTT GGT CAC ATA GTC TTC AGC GGG AAT GTT GCC C |  |  |
| NonChi-#1-F | GGG CAA CAT TCC CGC GAG AAG CCA TGT GAC CAA GTA CCT G |  |  |
| NonChi-#1-R | CAG GTA CTT GGT CAC ATG GCT TCT CGC GGG AAT GTT GCC C |  |  |
| NonChi-#2-F | GGG CAA CAT TCC CGC GTC AAG CCA TGT GAC CAA GTA CCT G |  |  |
| NonChi-#2-R | CAG GTA CTT GGT CAC ATG GCT TGA CGC GGG AAT GTT GCC C |  |  |
| NonChi-#3-F | GGG CAA CAT TCC CGC GTC ATG CCA TGT GAC CAA GTA CCT G |  |  |
| NonChi-#3-R | CAG GTA CTT GGT CAC ATG GCA TGA CGC GGG AAT GTT GCC C |  |  |
| NonChi-#4-F | GGG CAA CAT TCC CGC GAG ATC CTA TGT GAC CAA GTA CCT G |  |  |
| NonChi-#4-R | CAG GTA CTT GGT CAC ATA GGA TCT CGC GGG AAT GTT GCC C |  |  |
| NonChi-#5-F | GGG CAA CAT TCC CGC GTC AAC CTA TGT GAC CAA GTA CCT G |  |  |
| NonChi-#5-R | CAG GTA CTT GGT CAC ATA GGT TGA CGC GGG AAT GTT GCC C |  |  |
| NonChi-#6-F | GGG CAA CAT TCC CGC GTC ATG CTA TGT GAC CAA GTA CCT G |  |  |
| NonChi-#6-R | CAG GTA CTT GGT CAC ATA GCA TGA CGC GGG AAT GTT GCC C |  |  |
|  |  |  |  |

*Original and modified Chi sequences are underlined.
