# Supplementary material for: Bacterial RecA Protein Promotes Adenoviral Recombination during In Vitro Infection
Source: mSphere. 2018 Jun 20;3(3):e00105-18. doi: 10.1128/mSphere.00105-18 (PMC6010623; doi:10.1128/mSphere.00105-18)
Supplement: FIG S4 [file sph003182573sf4.pdf]

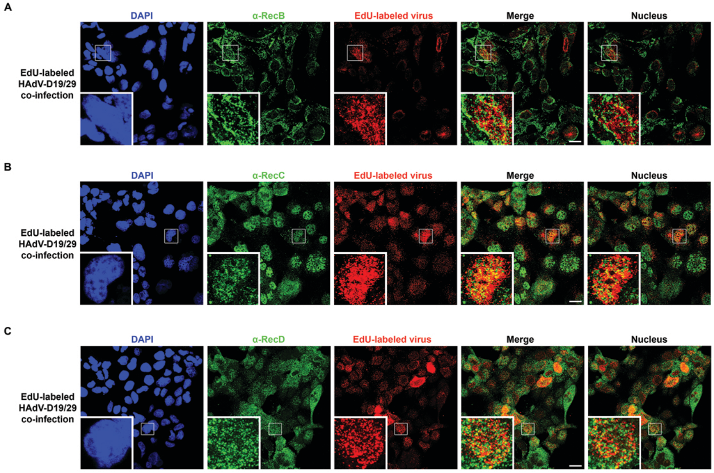
A
DAPI
α-RecB
EdU-labeled virus
Merge
Nucleus
EdU-labeled
HAdV-D19/29
co-infection
B
DAPI
α-RecC
EdU-labeled virus
Merge
Nucleus
EdU-labeled
HAdV-D19/29
co-infection
C
DAPI
α-RecD
EdU-labeled virus
Merge
Nucleus
EdU-labeled
HAdV-D19/29
co-infection
